# The HtrA-Like Serine Protease PepD Interacts with and Modulates the *Mycobacterium tuberculosis* 35-kDa Antigen Outer Envelope Protein

**DOI:** 10.1371/journal.pone.0018175

**Published:** 2011-03-22

**Authors:** Mark J. White, John P. Savaryn, Daniel J. Bretl, Hongjun He, Renee M. Penoske, Scott S. Terhune, Thomas C. Zahrt

**Affiliations:** 1 Department of Microbiology and Molecular Genetics, Medical College of Wisconsin, Milwaukee, Wisconsin, United States of America; 2 Center for Infectious Disease Research, Medical College of Wisconsin, Milwaukee, Wisconsin, United States of America; 3 Biotechnology and Bioengineering Center, Medical College of Wisconsin, Milwaukee, Wisconsin, United States of America; University of Osnabrueck, Germany

## Abstract

*Mycobacterium tuberculosis* remains a significant global health concern largely due to its ability to persist for extended periods within the granuloma of the host. While residing within the granuloma, the tubercle bacilli are likely to be exposed to stress that can result in formation of aberrant proteins with altered structures. Bacteria encode stress responsive determinants such as proteases and chaperones to deal with misfolded or unfolded proteins. *pepD* encodes an HtrA-like serine protease and is thought to process proteins altered following exposure of *M. tuberculosis* to extra-cytoplasmic stress. PepD functions both as a protease and chaperone *in vitro*, and is required for aspects of *M. tuberculosis* virulence *in vivo. pepD* is directly regulated by the stress-responsive two-component signal transduction system MprAB and indirectly by extracytoplasmic function (ECF) sigma factor SigE. Loss of PepD also impacts expression of other stress-responsive determinants in *M. tuberculosis*. To further understand the role of PepD in stress adaptation by *M. tuberculosis*, a proteomics approach was taken to identify binding proteins and possible substrates of this protein. Using subcellular fractionation, the cellular localization of wild-type and PepD variants was determined. Purified fractions as well as whole cell lysates from *Mycobacterium smegmatis* or *M. tuberculosis* strains expressing a catalytically compromised PepD variant were immunoprecipitated for PepD and subjected to LC-MS/MS analyses. Using this strategy, the 35-kDa antigen encoding a homolog of the PspA phage shock protein was identified as a predominant binding partner and substrate of PepD. We postulate that proteolytic cleavage of the 35-kDa antigen by PepD helps maintain cell wall homeostasis in *Mycobacterium* and regulates specific stress response pathways during periods of extracytoplasmic stress.

## Introduction

Tuberculosis remains a significant global health concern with estimates indicating that one-third of the world's population is currently latently infected by the causative organism, *Mycobacterium tuberculosis* [1]. The genetic programs required by *M. tuberculosis* for establishment, maintenance, and/or reactivation from persistent infection within the host remain poorly defined, but are thought to include stress-adaptation systems such as extracytoplasmic function (ECF) sigma factors and two-component signal transduction systems. *mprAB* is one of 11 complete two-component system encoded within the genome of *M. tuberculosis* [2]. This system directly regulates expression of numerous stress-responsive determinants in *M. tuberculosis* including ECF sigma factors *sigE* and *sigB*, alpha crystallin gene *acr2*, and serine protease *pepD* [3], [4], [5], [6]. MprAB is required for *in vivo* growth of the tubercle bacillus during persistent stages of infection [7], and its expression is up-regulated within an artificial granuloma model system [8] and under various conditions *in vitro* likely to be experienced by *M. tuberculosis* during residence within the granuloma [4], [6], [9].

PepD is a member of the HtrA-like protease family and is encoded immediately downstream of *mprAB* in all *Mycobacterium* species examined to date. HtrA-like proteases represent a well-conserved family of enzymes, and are responsible for degrading or refolding protein substrates following exposure to stress [10]. *In vitro*, PepD functions as both a protease and a chaperone [11]. In *Mycobacterium smegmatis*, loss of *pepD* enhances sensitivity of this bacterium to various cell wall-targeting antibiotics and detergents [12]. In contrast, *pepD* mutants of *M. tuberculosis* display a pleiotrophic phenotype; they are unaltered in survival following exposure to SDS [12], and they exhibit similar *in vivo* growth kinetics within tissues of infected mice compared to their wild-type counterparts [11]. However, these mutants do display an increased time to death in mice and are associated with reduced tissue pathology [11]. These phenotypes, coupled with the observation that *pepD* deletion results in upregulation of numerous stress-responsive determinants in *M. tuberculosis* under physiological conditions including *sigE* [12], underscores the complex regulation and multifaceted activity of this protein.

PepD is 464 amino acids and contains an N-terminal cytoplasmic domain (amino acids 1–101), a small transmembrane domain (amino acids 102–124), a catalytic protease domain (amino acids 166–364), and a C-terminal PDZ domain (amino acids 368–446). While most HtrA-like proteins possess either one or two PDZ domains [10] many of these proteins lack an N-terminal cytoplasmic domain. Previous studies have demonstrated that PepD processes artificial substrates including β-casein [11], [12] and pig heart citrate synthase [11]; however, natural substrates of PepD have yet to be identified. Proteolysis of β-casein requires the PDZ domain [12] and the catalytic serine at position 317 [11], [12]. The PDZ domain is also critical for regulating the activities of other HtrA proteases including DegS in *Escherichia coli*, one of the best characterized family members [13], [14], [15]. Additionally (or alternatively), interactions with PepD may localize to the N-terminal 101 amino acids, a region predicted to be cytoplasmic. To further understand the role of PepD in adaptation to stress, a proteomics approach was taken to identify proteins involved in the PepD-mediated stress response. Here we identify the 35-kDa antigen of *M. tuberculosis* (Rv2744c) as a target of the PepD protease.

## Methods

### Bacterial strains, media, and growth conditions

Strains and plasmids used in the study are described in Table S1. *Escherichia coli* Top 10 (Invitrogen, Carlsbad, CA), XL 10-Gold (Agilent Technologies, Santa Clara, CA), and DH5α were used for cloning procedures. BL21(DE3)/pLysS (Novagen, La Jolla, CA) was used to express and purify recombinant proteins in *E. coli*. All *E. coli* strains were grown with aeration at 37°C in Luria-Bertani (LB) broth or on LB agar (Thermo Fisher Scientific, Waltham, MA). When required, medium was supplemented with 25 µg/ml chloramphenicol (Sigma, St. Louis, MO), 150 µg/ml hygromycin B (AG Scientific, San Diego, CA), 100 µg/ml ampicillin (Thermo Fisher Scientific, Waltham, MA), and/or 50 µg/ml kanamycin sulfate (Thermo Fisher Scientific, Waltham, MA). *Mycobacterium* strains used in this study are all derivatives of *Mycobacterium tuberculosis* H37Rv (ATCC 27294) or *Mycobacterium smegmatis* mc^2^155 (ATCC 700084). Mycobacteria were grown with aeration at 37°C in Middlebrook 7H9 broth or 7H10 agar medium (Difco, Franklin Lakes, NJ) supplemented with 0.5% glycerol, 10% ADC or OADC (Difco, Franklin Lakes, NJ), and 0.05% Tween 80. For protein production, Mycobacteria were also grown in glycerol alanine salts (GAS) [16]. When required, Mycobacteria medium was supplemented with 25 µg/ml kanamycin sulfate (Thermo Fisher Scientific, Waltham, MA), 50 µg/ml hygromycin B (AG Scientific, San Diego, CA), and/or 50 µg/ml cyclohexamide (Thermo Fisher Scientific, Waltham, MA).

### DNA manipulations

Restriction enzyme digests, cloning, subcloning, and DNA electrophoresis were done according to standard techniques [17]. Oligonucleotides and primers were synthesized by Eurofins MWG Operon (Huntsville, AL) and are listed in Table S2. PCR was performed using High Fidelity Platinum PCR Supermix or Taq polymerase (Invitrogen, Carlsbad, CA). All amplified products were cloned into pCR2.1-TOPO (Invitrogen, Carlsbad, CA) and sequenced to confirm the absence of mutations. Ligations were performed using the Quick Ligation Kit (New England Biolabs, Beverly, MA) or T4 DNA ligase (Invitrogen, Carlsbad, CA). When necessary, plasmid DNA was treated with Antarctic phosphatase (New England Biolabs, Beverly, MA) to prevent religation of vector ends. Electroporation or transformation of plasmid DNA into *E. coli* or *Mycobacterium* was conducted as previously described [18]. Plasmid DNA was prepared using the QIAprep Spin Miniprep Kit (Qiagen, Venlo, The Netherlands) as recommended by the manufacturer. Genomic DNA was isolated from *M. tuberculosis* as described [18]. DNA fragments were purified using either the QIAquick Gel Extraction Kit or QIAquick PCR Purification Kit (Qiagen, Venlo, The Netherlands). The *pepD*S317A mutant allele was generated using the QuickChange XL site-directed mutagenesis kit (Stratagene, La Jolla, CA). DNA sequencing was performed with an ABI PRISM BigDye Terminator Cycle Sequencing Ready Reaction Kit (Applied Biosystems, Carlsbad, CA) using an automated long capillary method (ABI PRISM 3100 Genetic Analyzer, Applied Biosystems, Carlsbad, CA).

### Construction of epitope-tagged fusion proteins

Construction of pTZ758 encoding PepDΔTM was described previously [12]. Mycobacterium proteins containing N-terminal 3x-FLAG and C-terminal 6x-His epitope tags were generated using a three step cloning strategy. First, complementary oligonucleotides carrying the coding sequence for 3xFLAG were hybridized and ligated into cloning vector pCR2.1-TOPO. Second, the 3x-Flag coding sequence was amplified from pTZ806 using primers FLAGfwd-NdeI and FLAGrev-NdeI and subcloned into pET-24b (Novagen, La Jolla, CA) upstream of and in frame with the C-terminal 6x-His epitope tag. The resulting construct, pTZ842, served as the base plasmid for introduction of all subsequent *M. tuberculosis* sequences. Finally, to express epitope-tagged proteins in *Mycobacterium*, coding sequences were PCR amplified from pTZ842 variants using primers pET24fwd-PstI and pET24rev-HindIII and directionally subcloned into pSE100 [19]. This vector is an *E. coli-Mycobacterium* shuttle plasmid that contains the highly expressed *myc* promoter element upstream of the Tet operator site.

### Production and purification of recombinant proteins in *E. coli*

*E. coli* BL21(DE3)/pLysS strains containing over-expression constructs were grown overnight on selective LB agar medium, suspended in LB broth containing kanamycin and chloramphenicol, and grown to mid-exponential phase. Protein over-production was induced by the addition of 0.1 mM IPTG (Isopropyl-β-D-thiogalactopyranoside; Invitrogen, Carlsbad, CA) for 3 hours at 30°C. Induced cells were suspended in lysis buffer (20 mM Tris [pH 7.9], 500 mM NaCl, 5 mM imidazol, and 6 µg of DNAse/ml), passed 3× through a French press, and centrifuged at 25,000× *g* for 30 min. Cellular supernatants were passed over a nickel nitrilotriacetic acid-agarose column (Qiagen, Valencia, CA) and collected fractions pooled. Some proteins were further purified by size exclusion and anion exchange chromatography essentially as described [12]. Purified protein fractions were pooled and dialyzed overnight against dialysis buffer (20 mM Tris [pH 7.9], 150 mM NaCl, 20% glycerol). Purified proteins were stored at −80°C.

### Expression, fractionation, and localization of endogenous or epitope-tagged proteins in *Mycobacterium*

*M. smegmatis* or *M. tuberculosis* wild-type or recombinant derivatives containing pSE100-based expression constructs were grown in GAS medium to stationary phase (OD_600_>1.5). Culture filtrate proteins (CFP) were collected by passing spent broth medium through 0.25 µm low-protein binding filters (Corning, Lowell, MA) and concentrating the eluent using Amicon Ultracell-15 (10,000 molecular weight cutoff) spin columns. Non-secreted proteins were obtained by suspending the bacterial pellet in phosphate-buffered saline (PBS) containing protease inhibitors and mechanically disrupting the bacteria by bead beating. A low speed spin (11,000× *g*) was used to separate cell debris from the whole cell lysate. The resulting supernatant was clarified by passage through a 0.25 µm low-protein binding syringe filter (Corning, Lowell, MA). For all preparations generated in *M. tuberculosis*, protein-containing samples were checked for sterility using viability assays prior to removing from the BSL3 laboratory. Whole cell lysates were further separated into cell wall, cell membrane, and cytosolic fractions by differential ultracentrifugation as described previously [20]. Protein concentrations were determined using the BCA Protein Assay (Pierce, Rockford, IL). Protein lysates were added to 2× SDS-PAGE loading dye, boiled for 5 min, separated on 12% SDS-PAGE gels, and transferred onto Immobilon-P membranes (Millipore, Billerica, MA). Membranes were blocked in TTBS (20 mM Tris-HCl [pH 7.5], 500 mM NaCl, 0.5% Tween 20) containing 5% skim milk for at least 1 hour and probed with the antisera diluted in TTBS overnight at 4°C. Anitsera included rabbit polyclonal anti-PepD (1∶10,000 dilution) [12], rabbit polyclonal anti-MprA (1∶10,000 dilution; Covance, Princeton, NJ), rabbit polyclonal anti-MprB (1∶10,000 dilution; Covance, Princeton, NJ), rabbit polyclonal anti-*Mycobacterium tuberculosis* strain H37Rv LAM ([1∶10,000 dilution; NR-18321] from the TB Vaccine Testing and Research Materials Contract (BEI Resources, Manassas, VA)), and murine monoclonal anti-*Mycobacterium smegmatis* LAM ([1∶10,000 dilution; NR-13798] from the TB Vaccine Testing and Research Materials Contract (BEI Resources, Manassas, VA)). Membranes were washed in TTBS and incubated for 30 min at room temperature with goat anti-rabbit (1∶5,000 dilution) or goat anti-mouse (1∶5,000) secondary antibody conjugated to horseradish peroxidase (Pierce, Rockford, IL). Blots were developed using the SuperSignal West Femto Chemiluminescent Substrate kit (Pierce, Rockford, IL) and visualized on CL-XPosure X-ray film (Thermo Scientific, Rockford, IL).

### Immunoprecipitation of 3x-Flag-tagged proteins in *Mycobacterium* and identification of protein-protein interactions

*Mycobacterium* protein factions were collected from experimental or control strains expressing 3x-FLAG-tagged proteins as previously described. A 50 µl slurry of washed Anti-FLAG M2 agarose beads (Sigma, St. Louis, MO) was added to each sample and rocked at 4°C for 2 h. Beads were washed twice with PBS/0.1% Triton X-100 (Sigma, St. Louis, MO), and proteins eluted using 90 µM FLAG peptide (Sigma, St. Louis, MO) suspended in PBS/0.1% Triton X-100. Immunoprecipitated proteins were separated on 12% SDS-PAGE gels and were stained with GelCode Blue (Thermo, Waltham, MA) or by silver staining. Alternatively, proteins were separated and visualized using the Agilent 2100 Bioanalyzer and High Sensitivity Protein 250 Kit (Agilent Technology, Santa Clara, CA). For analysis by LC-MS/MS, protein samples were briefly run into the resolving gel to separate out the faster-migrating FLAG peptide. Remaining protein was excised from the gel, divided into two parts, and subsequently processed as two individual samples. The gel in each sample was diced into approximately 1 mm^3^ squares and destained using iterations of 25 mM ammonium bicarbonate (ABC)/50% acetonitrile (ACN) with agitation at 4°C. Gel pieces were then dehydrated with 100% ACN with subsequent rehydration and overnight incubation with 150 ng mass spectrometry grade Trypsin Gold (Promega, Madison, WI) in 50 mM ABC/10% ACN at 37°C with gentle rocking. Peptides were extracted using POROS 20 R2 reverse phase resin (Applied Biosystems, Foster City, CA) in 2.5% formic acid (FA)/0.1% trifluoroacetic acid (TFA) with agitation overnight at 4°C. Peptides were desalted using C18 ZipTip columns (Millipore, Billerica, MA) by washing with 0.1% TFA and eluting with 95% ACN/0.005% TFA followed by 70% ACN/0.03% TFA. Eluates from the same original resolving gel lane were combined and dried by spin vacuum evaporation. Peptides were resuspended in 5% ACN/0.1% FA, and loaded onto an ESI-LTQ XL mass spectrometer (Thermo, Waltham, MA) for identification by LC-MS/MS of the 6 most abundant peptides per precursor scan. Peptide identification was carried out using the search algorithm SEQUEST [21] by searching the *M. smegmatis* or *M. tuberculosis* protein database. Data was analyzed using the in-house software Visualize (http://proteomics.mcw.edu/visualize).

### Bacterial two-hybrid and β-galactosidase analysis

Specific protein-protein interactions were detected using the bacterial two-hybrid system, BACTH (Euromedex, Souffelweyersheim, France), per the manufacturer's directions. Briefly, the *Rv2744c* coding sequence was amplified from *M. tuberculosis* H37Rv genomic DNA and subcloned into T25- and/or T18-fusion vectors supplied by the manufacturer. Vectors constructed for this analysis are listed in Table S1. Plasmids were co-transformed into the *E. coli* BTH101 reporter strain. Resulting transformants were then plated on MacConkey/maltose agar medium, incubated at 30°C for 48–72 hours, and screened for the presence of a red colony phenotype indicative of protein-protein interactions. The extent of protein-protein interactions were quantified using β-galactosidase assays essentially as described [22].

### Protease activity assays

Proteolytic activity of PepDΔTM against potential substrates was determined using previously established procedures [12]. Briefly, increasing amounts of purified PepDΔTM were incubated with fixed amounts of the substrate in 50 mM potassium phosphate buffer (pH 7.5) at 37°C. Reaction mixtures were then separated using SDS-PAGE and stained with Coomassie blue for visualization.

### *In vitro* sensitivity assays

Sensitivity of *M. smegmatis* derivatives to antibiotic stress was measured using disc diffusion assays. Petri dishes were poured using 25 ml 7H9 bottom agar (1.2% agar) per dish. 100 µl aliquots of *M. smegmatis* cultures were added to 3.5 ml of 0.6% 7H9 top agar tempered to 55°C, poured onto bottom agar plates, and allowed to solidify. Sterile filter discs (6 mm) were added on top of the media and impregnated with 5 µl of 5 mg/ml vancomycin (Sigma, St. Louis, MO), 50 mg/ml cycloserine (Sigma, St. Louis, MO), 50 mg/ml isoniazid (Sigma, St. Louis, MO), or 10 µl of 50 mg/ml cefuroxime (Sigma, St. Louis, MO). Plates were incubated at 37°C and zones of inhibition were measured after 2 days. Stock and working antibiotic concentrations were prepared in sterile water.

### Statistical analysis

All statistical analyses were conducted using a Student's t-test. Values were determined to be statistically significant at *P*<0.05.

## Results

### Localization of PepD

**Figure 1:**
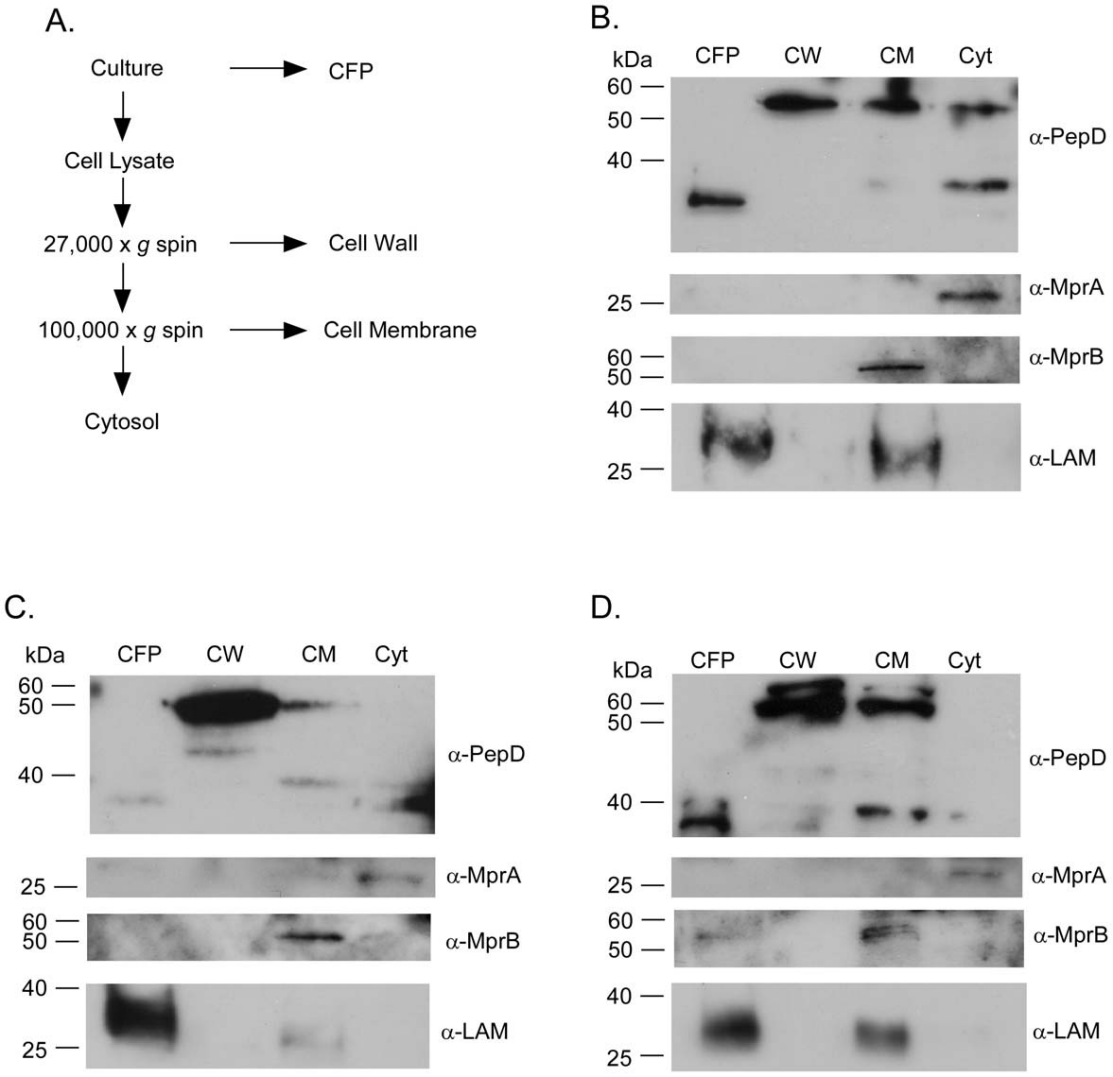
Localization of PepD. A) Schematic depicting the differential centrifugation protocol used to fractionate PepD in *M. tuberculosis*. B) Western blot showing the localization pattern of wild-type PepD in various subcellular compartments. MprA is used as a cytosolic marker, MprB is a cell membrane marker, and LAM is a cell envelope marker. C) Western blot showing the localization of the S317A mutant of PepD. D) Western blot demonstrating the localization of an overexpressed 3xFLAG-PepD-6xHis variant. Lanes: CFP, culture filtrate protein; CW, cell wall; CM, cell membrane; Cyt, cytosol.

PepD contains a single transmembrane domain and is predicted to localize to the plasma membrane. However, recent reports from us and others have indicated that PepD may also undergo autocatalysis and be secreted into the CFP [11], [12], [23]. To investigate PepD localization, subcellular compartments of *M. tuberculosis* Δ*pepD* expressing wild-type, mutant, or epitope-tagged forms of *pepD* (Table S1) were collected (Figure 1A) and subjected to immunoblot analyses [20]. *M. tuberculosis* Δ*pepD* expressing wild-type *pepD* from an integrated vector was observed as a ∼55 kDa protein in multiple fractions including the cytosol, cell membrane, and cell wall (Figure 1B). Additional lower molecular mass immunoreactive proteins were also observed in these fractions, likely a result of PepD autocatalysis during fractionation of the whole cell lysate as these peptides were less abundant in *M. tuberculosis* Δ*pepD* expressing the *pepDS317A* allele (Figure 1C). A processed form of PepD with a reduced molecular mass of ∼35 kDa was also observed in the CFP (Figure 1B), similar to that previously reported [23]. Observed proteins were PepD-specific, as immunoreactive proteins were not detected in fractions prepared from an *M. tuberculosis* Δ*pepD* strain (data not shown, and [12]). Control proteins including response regulator MprA, sensor kinase MprB, and glycolipid lipoarabinomannam localized to the expected compartments, validating the differential separation procedure (Figure 1B).

It has been reported that the 35-kDa form of PepD observed in the CFP results from a(n) autoproteolytic cleavage event(s) in the extracytoplasmic portion of the protein and is dependent on catalytic residue Ser-317 [11]. To determine whether this cleavage event was required for translocation of PepD from the cell membrane/cell wall to the CFP, analogous subcellular fractionation studies were carried out using *M. tuberculosis* Δ*pepD* expressing *pepDS317A* which encodes a PepD variant exhibiting 10% of the activity of wild-type PepD [11], [12]. In contrast to wild-type PepD, full length PepDS317A localized predominantly to the cell wall, although protein was also detected in the cell membrane and cytoplasm. Importantly, substantially less of the 35-kDa form of PepD was observed in the CFP, indicating that amino acid residue S317 is required for efficient processing of full length PepD to the 35-kDa form (Figure 1C). Finally, to confirm that over-production and/or addition of N- or C-terminal epitope tags does not alter the localization of PepD, fractionation studies were repeated using *Mycobacterium* Δ*pepD* strains carrying pTZ1049 (a pSE100 derivative constitutively expressing 3x-FLAG-PepD_WT_-6xHis). Epitope-tagged PepD over-produced in *M. tuberculosis* (Figure 1D) and *M. smegmatis* (Figure S1) fractionated similarly to wild-type PepD. Taken together, these data indicate that PepD traffics from the cytoplasm through the cell membrane to the cell wall where it is autoprocessed and eventually shed into the CFP as a 35-kDa form.

### Identification of PepDS317A interacting proteins

**Figure 2:**
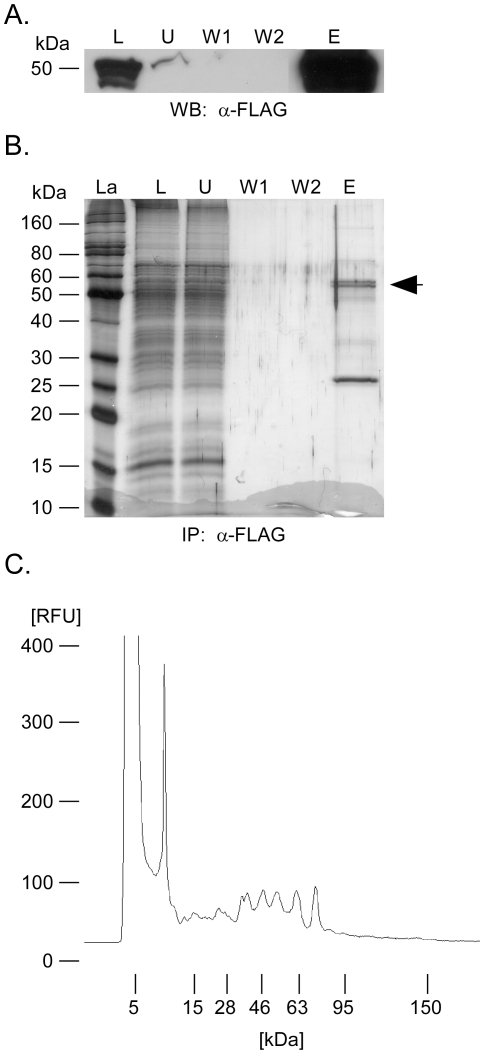
Immunoprecipitation of rPepD. Immunoprecipitation with anti-Flag antibody of *M. smegmatis* cultures expressing a vector containing 3xFlag-PepDS317A-6xHis. Immunoprecipitations were visualized using Western blot (A) or silver stain (B). Lanes: La, ladder; L, load; U, unbound; W1, first wash; W2, second wash; and E, elute. The arrow indicates the predicted location of 3xFlag-PepDS317A-6xHis. C) Electrophoretogram depicting the molecular masses and relative abundance of proteins co-immunoprecipitated with 3xFlag-PepDS317A-6xHis.

To identify potential interactants and/or substrates of PepD, co-immunoprecipitation (co-IP) studies were conducted with *M. smegmatis* Δ*pepD* carrying pSE100 or *M. smegmatis* Δ*pepD* expressing PepDS317A containing a 3xFLAG epitope on the amino terminus and a 6xHis sequence on the carboxyl terminus (pTZ1066). The PepDS317A variant was chosen to minimize autocatalysis and maximize protein-protein interactions with potential partners. In addition, both whole cell lysates and cell wall fractions were collected and incubated with anti-FLAG antibody conjugated to agarose beads to capture proteins from the various subcellular compartments that may interact with PepD. To determine the efficiency and specificity of IP, aliquots from whole cell lysates corresponding to the load (L), unbound (U), wash (W1 and W2), and elute (E) fractions were first analyzed and quantified for PepD by immunoblot using a mouse anti-FLAG monoclonal antibody. An immunoreactive protein migrating at the expected molecular mass was detected in samples prepared from *M. smegmatis* containing pTZ1066 (Figure 2A) but not pSE100 (data not shown). Quantification by densitometry indicated that ∼20% of the total epitope tagged PepDS317A present was pulled down (Figure 2A). Importantly, little PepDS317A was lost during washing and PepDS317A was enriched in the IP eluate (Figure 2A). When analyzed on a 12% SDS-PAGE gel stained with silver, a number of co-immunoprecipitating peptides were also apparent in addition to PepDS317A (Figure 2B). Analysis of IP eluants using an Agilent Bioanalyzer indicated that PepD interacted with multiple proteins possessing a range of molecular masses and that were present at various levels of abundance (Figure 2C). Taken together, these results demonstrate that PepDS317A interacts with numerous proteins in *M. smegmatis*.

To determine the identity of proteins co-immunoprecipitating with epitope-tagged PepDS317A, total cell or cell wall IP eluents from *M. smegmatis* Δ*pepD* containing pSE100 or pTZ1066 were subjected to LC-MS/MS analysis. Proteins were designated putative binding partners if they met the following criteria: (i) possessed a minimum peptide probability cut-off of 0.85, (ii) were represented by two or more unique peptides, and (iii) were present in the experimental pTZ1066 but not the control pSE100 dataset. 197 proteins were identified in the total cell lysate that co-immunoprecipitated with PepDS317A (Table S3), while 126 proteins from the cell wall fraction co-immunoprecipitated with this derivative (Table S4). 56 of the identified proteins were conserved in both *M. smegmatis* data sets (Table S5). These proteins fell into multiple functional categories including protein synthesis, energy metabolism, and protein fate (Table S5). The highest scoring protein based on percent coverage in both data sets was MSMEG_2695 (Tables S3 and S4). This protein is 91% similar and 85% identical to *M. tuberculosis* Rv2744c, and is also known as the 35-kDa antigen. Rv2744c is homologous to PspA from *E. coli*, a protein involved in the phage shock response in Gram-negative bacteria, and in homeostasis of the cell membrane including maintenance of proton motive force [24]. To determine whether the identified interactions were specific to only *M. smegmatis*, co-IP and LC-MS/MS analyses were repeated on total cell lysates isolated from *M. tuberculosis* Δ*pepD* carrying pSE100 or pTZ1066. A total of 200 proteins co-immunoprecipitated exclusively with PepDS317A in these samples (Table S6). Similar to *M. smegmatis*, the top scoring protein based on protein coverage was the 35-kDa antigen Rv2744c (Table S6). A variety of other proteins from multiple functional families including intermediary metabolism and respiration, lipid metabolism, and cell wall processes were also identified. Twenty of the identified *M. tuberculosis* proteins were also present in the list of 56 proteins conserved in both *M. smegmatis* datasets (Table S5). Based on these results, we conclude that PepD interacts with numerous proteins in both *M. smegmatis* and *M. tuberculosis*, including the 35-kDa antigen.

### Identification of PepD substrates

To determine whether any of the *M. tuberculosis* proteins associated with PepD are potential substrates, IP eluants were incubated with active PepDΔTM [12], subjected to trypsin digestion and analyzed using LC-MS/MS. Peptides resulting solely from tryptic cleavage were removed from the dataset, leaving a collection of 13 unique proteins with semi-tryptic cleavage sites (Table S7). PepD was the highest scoring protein with 33 unique non-tryptic peptides (Table S8). Several of the identified cleavage sites match those previously reported for PepD which undergoes autocatalysis [11], validating the utility of this assay. Proteins not meeting the minimum peptide number and probability requirement described previously were further removed from the list, resulting in 4 candidate targets each consisting of a single semi-tryptic peptide fragment (Table 1). Based on protein probability, Rv2744c remained the top scoring protein (Table 1). Other proteins identified included Rv1310 (AtpD), Rv0350 (DnaK), and Rv1266c (PknH). Thus, PepD alone or as part of a larger protein complex interacts with and potentially cleaves several proteins in *M. tuberculosis*, including the 35-kDa antigen.

**Table 1:** Putative PepD substrates with semi-tryptic ends.

Rv No.^a^	Gene^a^	Protein Probability^b^	Peptide identified^c^	Gene Product^a^
Rv2744c		1.00	F.AAQLVTAEQSVEDLK	35-kDa protein
Rv1310	*atpD*	0.96	V.TGPVVDVEFPR	ATP synthase subunit beta
Rv1266c	*pknH*	0.89	A.GAAAVVLVLVLGAIGIWIAIR	Serine/threonine protein kinase
Rv0350	*dnaK*	0.89	P.DEVVAVGAALQAGVLK	Chaperone protein

a Protein information based on Pasteur Institutes' Tuberculist website (http://genolist.pasteur.fr/Tuberculist).

b Protein probability assigned by spectra following LC-MS/MS.

c Semi-tryptic peptide identification with annotated cleavage site.

### The 35-kDa antigen is a binding partner and potential substrate of PepD

**Figure 3:**
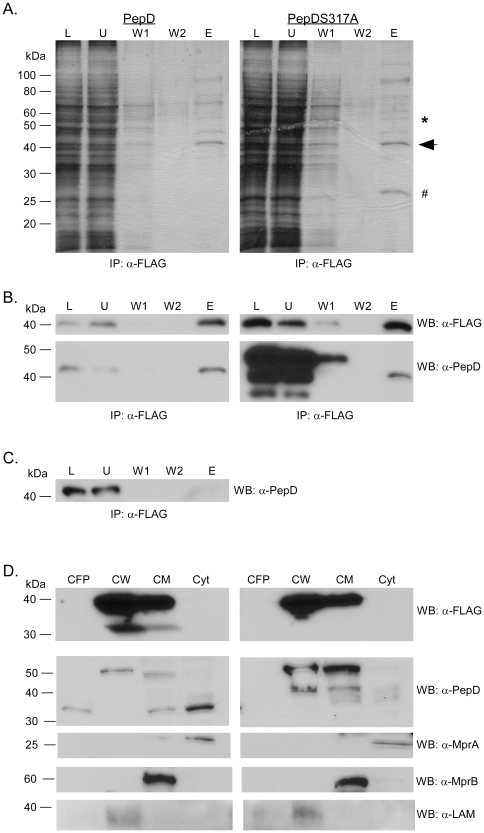
Immunoprecipitation of rRv2744c. (A) Immunoprecipitation with anti-Flag antibody of *M. smegmatis* Δ*pepD* strains complemented with either *M. tuberculosis pepD*_wt_ (left panel) or *pepD*317A (right panel) and expressing a vector containing 3xFlag-Rv2744c-6xHis. Immunoprecipitations were visualized by silver stain. Lanes: L, load; U, unbound; W1, first wash; W2, second wash; and E, elute. The arrow indicates the predicted location of 3xFlag-Rv2744c-6xHis, the asterisk (*) indicates the predicted location of PepD, and the hatch mark indicates the location of MSMEG_2695. (B) Western blot demonstrating the immunoprecipitation of PepD with 3xFlag-Rv2744c-6xHis using antibodies to either the Flag epitope or PepD. (C) Western blot demonstrating the specificity of anti-FLAG immunoprecipitation from a *M. smegmatis* Δ*pepD* strain carrying the pSE100 expression vector alone. (D) Western blot showing the localization of 3xFlag-Rv2744c-6xHis in various subcellular compartments in *M. smegmatis*. Lanes: CFP, culture filtrate protein; CW, cell wall; CM, cell membrane; Cyt, cytosol.

The 35-kDa antigen was previously identified to be upregulated upon exposure to vancomycin, implicating a role for this determinant in resistance to cell envelope stress [25]. To confirm that this protein interacted with PepD, reverse co-IP's were carried out in *M. smegmatis* Δ*pepD* strains carrying pSE100 or pTZ1175 (pSE100 expressing 3xFLAG-Rv2744c-6xHis). *M. smegmatis* Δ*pepD* strains utilized in this assay also expressed *M. tuberculosis pepD* or *pepD*S317A [12], as the polyclonal antibody to *M. tuberculosis* PepD does not recognize the homologue in *M. smegmatis*. SDS-PAGE analysis of anti-FLAG IP eluants indicated the presence of proteins migrating at molecular masses expected for both epitope-tagged Rv2744c and PepD (Figure 3A). Western blot analysis further confirmed the presence of these proteins in IP eluants from strains expressing Rv2744c (Figure 3B), but not the vector only control (Figure 3C). Interestingly, a protein running at the expected molecular mass of endogenous MSMEG_2695 was also observed in the *M. smegmatis* strain expressing *M. tuberculosis pepDS317A* but not in the strain expressing wild-type *M. tuberculosis pepD* (Figure 3A and Figure 2B). LC-MS/MS analysis of this protein confirmed it as MSMEG_2695 (data not shown).

To verify that the 35-kDa antigen localized to the same compartment(s) as PepD, fractionation and Western blot assays were carried out on *M. smegmatis* strains expressing epitope-tagged Rv2744c. The 35-kDa antigen localized to the cell wall and cell membrane compartments (Figure 3D), consistent with the localization pattern of PepD previously observed. Also, the presence of a lower molecular weight protein immunoreactive with the anti-FLAG monoclonal antibody was also present in the strain expressing wild-type *M. tuberculosis pepD* but not *pepDS317A* (Figure 3D), indicating that PepD potentially cleaves the 35-kDa antigen. While the size of this lower molecular weight band is not consistent with the cleavage site detected by LC-MS/MS (Table 1), the two experiments utilize different detection readouts and cannot be directly compared. Additionally, peptides from the C-terminal region of the 35-kDa antigen were not detected by LC-MS/MS for any of the *Mycobacterium* PepD immunoprecipitation experiments (Supplemental Tables S3 and S4), the region of Rv2744c predicted to be cleaved by PepD based on results from Western blot studies (Figure 3D). Regardless, this data confirms the interaction between the 35-kDa antigen and PepD, and further supports the contention that this protein is a substrate of PepD.

### Rv2744c over-production restores resistance to Δ*pepD* mutants of *M. smegmatis* against specific cell wall-damaging antibiotics

Previous studies have demonstrated that Δ*pepD* mutant strains of *M. smegmatis* exhibit increased sensitivity to cell wall antibiotics including cycloserine and cefuroxime [12]. To determine whether Rv2744c may function to regulate cell wall homeostasis in *Mycobacterium* [24], *in vitro* antibiotic susceptibility assays were carried out with Δ*pepD M. smegmatis* mutant strains expressing *Rv2744c*. Δ*pepD* strains harbouring pSE100 were significantly more sensitive to vancomycin, cycloserine, and cefuroxime compared to their wild-type counterparts containing pSE100 (Table 2). In contrast, expression of *Rv2744c* from pSE100 in *M. smegmatis* Δ*pepD* restored resistance to these antibiotics to near wild-type levels (Table 2). The observed sensitivity of *M. smegmatis* Δ*pepD* mutants but not Δ*pepD* mutants expressing *Rv2744c* was specific to antibiotics targeting the peptidoglycan, as no differences were observed between *M. smegmatis* strains following exposure to isoniazid, an antibiotic that inhibits fatty acid synthase and synthesis of cell wall mycolic acids (Table 2). Overall, these data argue that over-expression of *Rv2744c* increases resistance of *M. smegmatis* to peptidoglycan-disrupting antibiotics, possibly helping to maintain cell wall homeostasis.

**Table 2:** Susceptibility of *M. smegmatis* strains to cell-wall targeting antibiotics^a^.

Strain	Vancomycin^b^	Cycloserine^c^	Cefuroxime^d^	INH^e^
mc^2^155/pSE100	13.7±0.3	18.5±0.3	6±0	46.7±0.3
Δ*pepD*/pSE100	16±0*	20.5±0.3*	12.7±0.9*	46.7±0.3
Δ*pepD*/pTZ1175	14.3±0.3	18.8±0.2	8.3±0.3*	46.3±0.3

a Diameter of inhibition zones (mm) including disc diameter of 6 mm. Values are expressed as mean ± SEM.

b 5 µl of 5 mg/ml vancomycin.

c 5 µl of 50 mg/ml cycloserine.

d 10 µl of 50 mg/ml cefuroxime.

e 5 µl of 50 mg/ml isoniazid.

*, *P*<0.05 when compared to mc^2^155/pSE100.

## Discussion

*M. tuberculosis* must adapt to harsh environmental conditions within the host to successfully establish, maintain, or reactivate from acute and/or chronic infection states. As one part of its survival strategy, the bacterium encodes proteases and chaperones which act to either degrade and/or refold proteins whose structure has become altered following stress exposure. *Mycobacteria* encode homologs of the evolutionary conserved HtrA family of proteins, which have been shown in both Gram-negative and Gram-positive organisms to regulate adaptation to extracytoplasmic stress [26], [27], [28], [29]. In *M. tuberculosis*, the physiological role of HtrA-family proteins has remained largely undefined. Here, we provide further insight into one HtrA family member, PepD, which has been previously shown to impact the MprAB and SigE stress response networks in *M. tuberculosis in vitro* [12], and contribute to *M. tuberculosis* virulence *in vivo* [11].

While PepD is predicted to localize to the cell membrane in *M. tuberculosis*, subcellular fractionation studies carried out with *M. tuberculosis* and *M. smegmatis* expressing wild-type or epitope-tagged forms of PepD indicate that this protein localizes to multiple subcellular compartments, including the cell membrane, the cell wall, and the CFP. Interestingly, mutations to the catalytic serine at position 317 of PepD affect not only the ability of the protein to undergo autocatalysis [11], but also affect its pattern of localization and its ability to be secreted into the CFP. In contrast to wild-type PepD, PepDS317A is observed predominantly in the cell wall with little protein observed in the cell membrane and culture filtrate. The size of the PepD product secreted into the culture filtrate is consistent with the ∼35-kDa autoproteolytic peptide observed previously by LC-MALDI-MS and LC-ESI-MS *in vitro* with purified protein [11]. While this peptide presumably retains the catalytic and PDZ domains, it remains unclear whether this peptide has a biological function once secreted. We were unable to detect by immunoblot the 10-kDa autoproteolytic product previously reported by MohamedMohaideen *et al.* to contain the PDZ domain alone [11]. It is possible that this product may exhibit a short half-life, or may be further processed into a form that is outside the detection parameters used in these studies.

In an effort to delineate the specific mechanism by which PepD contributes to the *M. tuberculosis* stress response, a proteomic approach was used to identify proteins or protein complexes that interact with PepD. In *M. tuberculosis* or *M. smegmatis*, the most prominent PepD binding protein identified was the 35-kDa antigen, Rv2744c or MSMEG_2695, respectively. Bioinformatic analysis indicates that Rv2744c is a member of the PspA family of proteins. These proteins participate in the phage shock response that has been largely studied in Gram-negative bacteria where they are thought to participate in multiple functions. In bacteria, PspA is involved in maintaining the proton motive force [24], and it acts as a negative regulator of the *psp* operon [30], [31]. A PspA homolog in plants, VIPP1, is important in photosynthesis [32], [33]. In *M. tuberculosis*, *Rv2744c* lies in an operon with upstream transcription factor *clgR* and downstream gene *Rv2743c* encoding a predicted membrane protein [2]. ClgR regulates its own expression and several other genes in *M. tuberculosis* including proteases and chaperones involved in protein homeostasis [34]. ClgR may also regulate determinants involved in the maintenance of cellular redox potential and energy generation [35]. *clgR* is upregulated in *M. tuberculosis* following exposure to various extracytoplasmic stress including subinhibitory concentrations of vancomycin and thioridazine [25], [36]. Vancomycin interferes with peptidoglycan biosynthesis, and thioridazine is believed to inhibit efflux pumps in *M. tuberculosis* leading to a disruption in aerobic respiration [37], [38]. *clgR* is also upregulated following redox stress, heat shock, acid stress, and during intramacrophage growth [35]. Interestingly, *pepD* and *Rv2744c* are both regulated by SigE, suggesting these proteins may respond to similar stresses [39].

While both PepD and the 35-kDa antigen localize to the cell membrane and cell wall, the nature of their interaction remains unclear. Proteomic studies indicate that the 35-kDa antigen is a substrate for PepD proteolysis. A processed form of epitope tagged Rv2744c is present in cell wall and cell membrane fractions prepared from *M. smegmatis* Δ*pepD* strains expressing *M. tuberculosis pepD* but not the catalytic site mutant, *pepD*S317A (Figure 3D). Additionally, a protein corresponding to endogenous MSMEG_2695 is co-immunoprecipitated from whole cell lysates prepared from *M. smegmatis* Δ*pepD* strains expressing *M. tuberculosis pepD*S317A but not wild-type *M. tuberculosis pepD* (Figure 3A). However, we have been unable to demonstrate proteolysis of purified Rv2744c by PepDΔTM *in vitro* (data not shown). This could be due to a number of factors. It is possible that proteolysis requires involvement of an accessory protein or some other activating interaction, similar to what is seen with other HtrA family members [14], [15], [40]. Consistent with this possibility, LC-MS/MS data indicate that PepD potentially forms complexes with multiple proteins. Alternatively, it is possible that PepDΔTM is not capable of binding purified epitope-tagged Rv2744c or mediating its cleavage. Interestingly, Rv2744c seems to associate with a specific isoform of PepD that is slightly smaller than that predicted for the full-length protein (Figure 3B). Given that PepDΔTM lacks the cytoplasmic domain and transmembrane domain, it may be unable to assume the proper confirmation necessary for efficient Rv2744c interaction and/or cleavage. While we predict that the PDZ domain of PepD mediates protein interactions with the 35-kDa antigen, PepD also possesses a large cytoplasmic domain. A subset of proteins co-immunoprecipitating with PepD in both *M. tuberculosis* and *M. smegmatis* are predicted to localize to the cytoplasmic compartment, raising the possibility that additional interactions may be mediated through this domain. The HtrA-like protein Rv1223, which is predicted to be essential in *M. tuberculosis* [11], [12], also contains a large 175 amino acid cytoplasmic domain [2]; however, other HtrA-family proteins in *M. tuberculosis* and in other organisms lack such a domain. Therefore, further work is needed to delineate whether additional interactions within the bacterial cell cytoplasm are necessary for optimal autocatalysis or processing of substrates by PepD in the extracytoplasmic space.

In addition to the 35-kDa antigen, three other proteins were identified as potential substrates of PepD based on proteomic analyses. AtpD is an ATP synthase subunit involved in maintaining the proton motive force in Gram-positive bacteria [41], [42]. DnaK is an ubiquitous chaperone protein involved in the heat shock response [43], [44]. PknH is a membrane-associated serine/threonine kinase involved in signal transduction, and is necessary for arabinose metabolism [45]. The identified PepD cleavage site for PknH occurs near the transmembrane domain on the cytoplasmic face, a location unlikely to be accessible by the PepD protease domain. However, it is possible that PknH is cleaved by two separate proteases at the transmembrane interface in a fashion similar to RseB in *E. coli*. This process, termed Regulated Intramembrane Proteolysis (RIP), involves the activities of an HtrA-family protease, DegS, and a metalloprotease, RseP (YaeL) [26]. Because the extracytoplasmic side of the transmembrane domain of PknH contains an arginine and lysine, it is conceivable that PepD cleaves in this area and produces a peptide that was missed during our semi-tryptic mass spectrometric analysis. Alternatively, the peptide identified may be the product of a cleavage event mediated by another protease, as PepD was able to co-immunoprecipitate multiple proteases in both *M. tuberculosis* and *M. smegmatis*. Regardless, the identified binding proteins and substrates provide a starting point for further investigations into the physiological role of PepD in *M. tuberculosis*.

**Figure 4:**
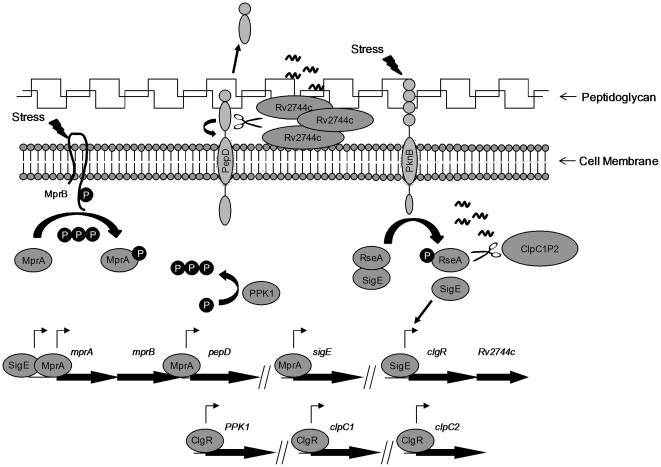
Model of Rv2744c regulation by PepD. Under stress conditions that alter cell wall peptidoglycan, PknB becomes activated and phosphorylates RseA. RseA phosphorylation leads to proteolytic degradation by the ClpC1P2 protease, releasing sigma factor SigE and activating gene determinants comprising the SigE regulon. SigE positively regulates *clgR*, *Rv2744c*, and *mprA*. ClgR autoregulates its own expression and that of downstream gene *Rv2744c*, and upregulates expression of other genes including *ppk1*, *clpC1*, and *clpC2*. MprA in turn upregulates *sigE* expression, leading to the direct and indirect upregulation of *pepD*. Rv2744c traffics to the cell membrane and cell wall where it may oligomerize and help maintain cell envelope homeostasis. PepD traffics to the same cellular compartment where it acts to maintain Rv2744c at appropriate levels. Cessation of peptidoglycan stress results in RseA stabilization, sequestration of SigE, and subsequent downmodulation of the SigE and MprA signaling pathways.

Based on this data, we postulate that PepD functions to proteolytically regulate Rv2744c levels to help maintain cell wall/cell envelope homeostasis in *M. tuberculosis* (Figure 4). A model is also proposed that builds upon observations previously reported by Barik *et al* [46] and others [3], [47], [48] concerning interactions between the SigE and MprAB signalling pathways in *M. tuberculosis* following exposure to extracytoplasmic stress. The serine/threonine protein kinase, PknB, contains PASTA domains that have been postulated to bind peptidoglycan and may serve as cell wall sensors [49]. As the peptidoglycan becomes disordered due to extracellular stress, PknB activates and phosphorylates RseA, the anti-sigma factor of SigE. Phosporylation of RseA leads to proteolytic degradation of this protein by ClpC1P2, releasing SigE and inducing expression of components of the SigE regulon including *mprA* and *clgR* [39], [46], [50]. MprA and ClgR in turn upregulate gene products within their cognate regulons including *clgR* itself, *clpC1*, *clpP2*, *ppk1*, *pepD*, and *sigE* [3], [34], [35]. Upregulation of *clp* genes initiates a positive feedback loop through SigE by enhancing degradation of RseA. Similarly, upregulation of *ppk1* encoding polyphosphate kinase increases polyphosphate levels and enhances activation of the MprAB two-component system [47], mediating a positive feedback loop through SigE [3]. The Rv2744c generated following upregulation of *clgR* is secreted extracytoplasmically, where it functions in an as-of-yet undefined role to help mediate resistance to the recognized stress. In *Escherichia coli* and other bacterial species, PspA forms higher order oligomers where the protein is thought to function as a structural scaffold to help maintain proton motive force [51], [52], [53], [54]. While it is currently unclear if higher order oligomers are formed by Rv2744c in *M. tuberculosis*, Rv2744c can interact with itself in bacterial two-hybrid assays carried out in *E. coli* (Figure S2). Over-production of Rv2744c and/or exposure of this protein to stress that perturbs the cell wall, including that mediated through peptidoglycan-disrupting agents, may lead to unstructured regions of Rv2744c that become recognized by PepD. Subsequent processing by PepD would help minimize the over-accumulation of Rv2744c in the cell wall/cell membrane. Alternatively, cleavage by PepD may be important for some aspect of Rv2744c function. Finally, it is also possible that cleavage of Rv2744c by PepD may represent a mechanism for terminating the membrane stress response following cessation of the inducing stimulus. While none of these possibilities are mutually exclusive, production of Rv2744c helps restore resistance of *M. smegmatis* Δ*pepD* strains to peptidoglycan-perturbing agents, allowing maintenance of cell wall homeostasis following exposure to extracytoplasmic stress. Future studies are aimed at delineating the specific mechanism by which Rv2744c participates in cell wall homeostasis, and defining the other factors that participate in this stress response pathway.

## Supporting Information

Figure S1 **Localization of 3xFLAG-PepD-6xHis in** ***M. smegmatis*** **.** Western blot demonstrating the localization of an overexpressed 3xFLAG-PepD-6xHis variant in *M. smegmatis* mc^2^155. Lanes: CFP, culture filtrate protein; CW, cell wall; CM, cell membrane; Cyt, cytosol.
(TIF)

Figure S2 **Quantification of Rv2744c interaction by bacterial two-hybrid assays.** *E. coli* BTH101 was transformed with various bacterial two-hybrid plasmids and subjected to β-galactosidase assays to quantify protein-protein interactions. pKT25 and pUT18 without inserts served as the negative control. pTZ1185 (pKT25 containing *Rv2744c*) and pTZ1182 (pUT18 containing *Rv2744c*) were used to investigate interaction of Rv2744c with itself. pKT25zip and pUT18Czip served as the positive control.
(TIF)

Table S1 Bacterial strains and plasmids used in this study.
(RTF)

Table S2 Oligonucleotides used in this study.
(RTF)

Table S3 Proteins from *M. smegmatis* whole cell lysate co-immunoprecipitating with 3xFLAG-PepDS317A-6xHis.
(XLSX)

Table S4 Proteins from *M. smegmatis* cell wall fraction co-immunoprecipitating with 3xFLAG-PepDS317A-6xHis.
(XLSX)

Table S5 Proteins identified in both *M. smegmatis* cell wall and whole cell lysate preparations that co-immunoprecipitate with 3x-FLAG-PepDS317A-6xHis.
(RTF)

Table S6 Proteins from *M. tuberculosis* whole cell lysate co-immunoprecipitating with 3xFLAG-PepDS317A-6xHis.
(XLSX)

Table S7 PepD proteolysis of *M. tuberculosis* proteins co-immunoprecipitating with 3xFLAG-PepDS317A-6xHis.
(XLSX)

Table S8 Identification of putative autolytic PepD cleavage sites using LC-MS/MS.
(RTF)
