# Supplementary figures and images for: The HtrA-Like Serine Protease PepD Interacts with and Modulates the Mycobacterium tuberculosis 35-kDa Antigen Outer Envelope Protein
Source: PLoS One. 2011 Mar 22;6(3):e18175. doi: 10.1371/journal.pone.0018175 (PMC3062566; doi:10.1371/journal.pone.0018175)

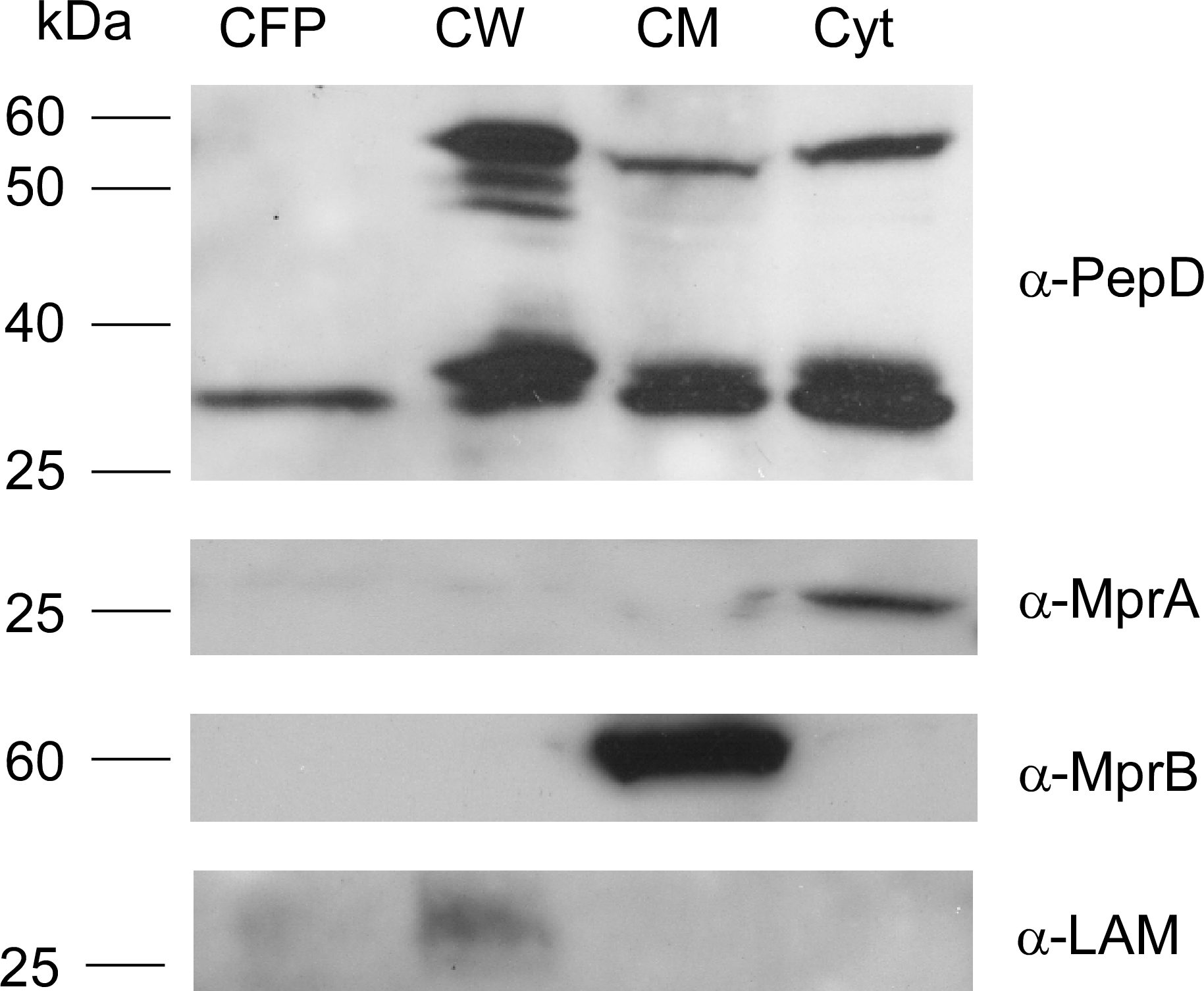

Supplement: Figure S1 — Localization of 3xFLAG-PepD-6xHis in M. smegmatis . Western blot demonstrating the localization of an overexpressed 3xFLAG-PepD-6xHis variant in M. smegmatis mc2155. Lanes: CFP, culture filtrate protein; CW, cell wall; CM, cell membrane; Cyt, cytosol. (TIF) [file pone.0018175.s001.tif]

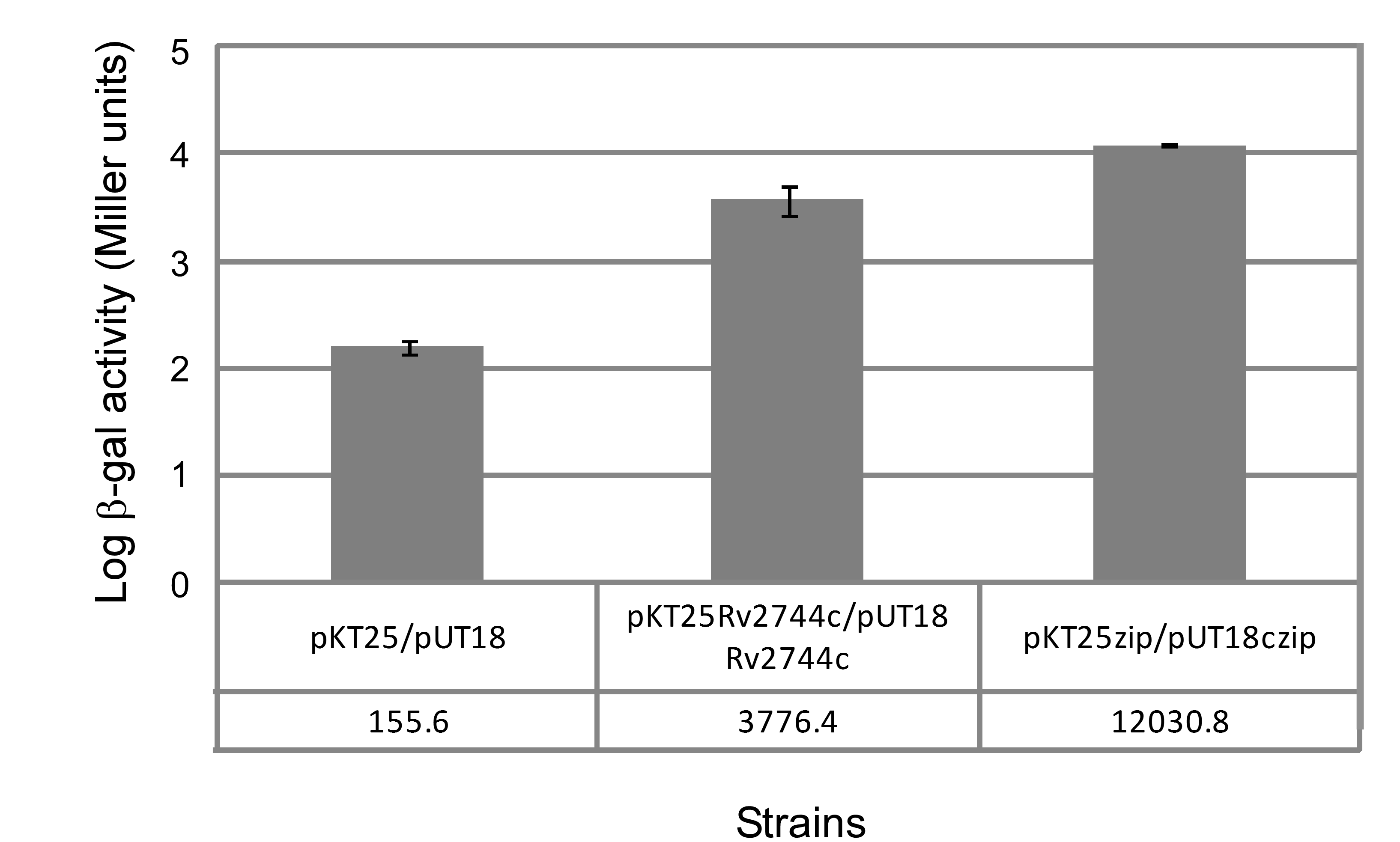

Supplement: Figure S2 — Quantification of Rv2744c interaction by bacterial two-hybrid assays. E. coli BTH101 was transformed with various bacterial two-hybrid plasmids and subjected to β-galactosidase assays to quantify protein-protein interactions. pKT25 and pUT18 without inserts served as the negative control. pTZ1185 (pKT25 containing Rv2744c) and pTZ1182 (pUT18 containing Rv2744c) were used to investigate interaction of Rv2744c with itself. pKT25zip and pUT18Czip served as the positive control. (TIF) [file pone.0018175.s002.tif]
