# Supplementary material for: The HtrA-Like Serine Protease PepD Interacts with and Modulates the Mycobacterium tuberculosis 35-kDa Antigen Outer Envelope Protein
Source: PLoS One. 2011 Mar 22;6(3):e18175. doi: 10.1371/journal.pone.0018175 (PMC3062566; doi:10.1371/journal.pone.0018175)
Supplement: Table S1 — Bacterial strains and plasmids used in this study. (RTF) [file pone.0018175.s003.rtf]

Table S1. Strains and plasmids used in this study


Strain/Plasmid	

Genotype or Description	

Application	

Reference or Source
	

STRAINS
				
M. tuberculosis H37Rv
	Laboratory strain		ATCC 27294	
TB22
	M. tuberculosis H37Rv  ÄpepD		(12)	
M. smegmatis mc2155
	Laboratory strain		ATCC 700084	
TCZ1392
	M. smegmatis mc2155 ÄpepD		(12)	
E. coli DH5á	F- ö80lacZÄM15 Ä(lacZYA-argF) U169 recA1 endA1 hsdR17 (rk-, mk+) phoA supE44 ë- thi-1 gyrA96 relA1
	Cloning	Lab collection	
E. coli TOP10	F- mcrA Ä(mrr-hsdRMS-mcrBC) ö80lacZÄM15 ÄlacX74 recA1 araD139 Ä(araleu)7697 galU galK rpsL (StrR) endA1 nupG
	Cloning	Invitrogen	
E. coli XL 10-Gold
	endA1 glnV44 recA1 thi-1 gyrA96 relA1 lac Hte Ä(mcrA)183 Ä(mcrCB-hsdSMR-mrr)173 tetR F'[proAB lacIqZÄM15 Tn10(TetR Amy CmR)]
	Cloning	Stratagene	
E. coli BL21(DE3)/pLysS
	F- ompT hsdSB(rB-mB-) gal dcm (DE3)/ pLysE (CamR)	Protein over-expression 	Novagen	
E. coli BTH101
	F- cya-99 araD139 galE15 galK16 rpsL1 (Strr) hsdR2 mcrA1 mcrB1	Bacterial two-hybrid	Euromedex	
				
PLASMIDS
				
pCR2.1-TOPO
	3.9-kb plasmid for cloning PCR products; Ampr, Kanr	PCR cloning vector	Invitrogen	
pET-24b	5.3-kb plasmid allowing C-terminal protein fusion to 6X-His; Kanr	Protein over-expression	Novagen
	
pKT25
	3.4-kb plasmid allowing N-terminal protein fusion to T25 fragment; Kanr	Two-hybrid vector	Euromedex	
pKNT25
	3.4-kb plasmid allowing C-terminal protein fusion to T25 fragment; Kanr	Two-hybrid vector	Euromedex	
pMV306	3.9-kb plasmid for single copy integration into mycobacterial chromosome; Kanr
	Complementation vector
	Lab collection	
pSE100
	5.5-kb plasmid allowing expression from a tet-inducible promoter	Protein over-expression
	(19)	

pUT18
	
3.0-kb plasmid allowing C-terminal protein fusion to T18 fragment; Ampr	
Two-hybrid vector	
Euromedex	
pUT18C
	3.0-kb plasmid allowing N-terminal protein fusion to T18 fragment; Ampr	Two-hybrid vector	Euromedex	
pTZ792
	pMV306 containing pepD and moaB2 coding sequences from M. tuberculosis; Kanr	Complementation	(12)
	
pTZ806
	pCR2.1-TOPO containing 3xFLAG sequence; Ampr, Kanr	Cloning	This study	
pTZ842
	pET-24b containing 3xFLAG sequence at NdeI site; Kanr
	Over-expression	This study	
pTZ903	pMV306 containing pepDS317A and moaB2 coding sequences; Kanr	Complementation	(12)
	
pTZ1025
	pTZ842 containing pepD coding sequence; Kanr	Over-expression	This study	
    pTZ1049
	pSE100 containing 3xFlag-pepD coding sequence; Hygr	Over-expression	This study	
pTZ1057
	pCR2.1 containing pepDS317A coding sequence; Ampr, Kanr	Over-expression	This study	
    pTZ1066
	pSE100 containing 3xFlag-pepDS317A coding sequence; Hygr	Over-expression	This study	
pTZ1168
	pET-24b containing Rv2744c coding sequence; Kanr	Over-expression	This study	
    pTZ1171
	pTZ842 containing Rv2744c coding sequence; Kanr	Over-expression	This study	
    pTZ1175
	pSE100 containing 3xFlag-Rv2744c coding sequence; Hygr	Over-expression	This study	
    pTZ1182
	pUT18 containing Rv2744c coding sequence; Ampr	Two-hybrid	This study	
    pTZ1183
	pUT18C containing Rv2744c coding sequence; Ampr	Two-hybrid	This study	
    PTZ1185
	pKT25 containing Rv2744c coding sequence; Kanr	Two-hybrid	This study	
