# Supplementary material for: The HtrA-Like Serine Protease PepD Interacts with and Modulates the Mycobacterium tuberculosis 35-kDa Antigen Outer Envelope Protein
Source: PLoS One. 2011 Mar 22;6(3):e18175. doi: 10.1371/journal.pone.0018175 (PMC3062566; doi:10.1371/journal.pone.0018175)
Supplement: Table S2 — Oligonucleotides used in this study. (RTF) [file pone.0018175.s004.rtf]

Table S2.  Oligonucleotides used in this study


Primers	

Descriptiona	

Application	

Source
	
				
M13rev
	5'-CAGGAAACAGCTATGAC-3'	Sequencing	Invitrogen	
    M13for
	5'-GTAAAACGACGGCCACT-3'	Sequencing	Invitrogen	
    pET15b-F
	5'-CCCGCGAAATTAATACGACTCACTATAGGG-3'	Sequencing	Eurofins MWG Operon	
pET15b-R
	5'-TTATGCTAGTTATTGCTCAGCGGTGGCAGC-3'	Sequencing	Eurofins MWG Operon	
pSE100F2
	5'-CGAGCGGGAGAACTCCCTATCAGT-3'	Sequencing	Eurofins MWG Operon	
pSE100R2
	5'-ATAACGTTCTCGGCTCGATGATCCC-3'	Sequencing	Eurofins MWG Operon	
3FLAG-F	5'-TATGGACTACAAGGACGACGATGACAAAGACTACAAGGACGACGATGACAAAGAC-TACAAGGACGACGATGACAAACA-3'
	Cloning	Eurofins MWG Operon	
3FLAG-R2	5'-TATGTTTGTCATCGTCGTCCTTGTAGTCTTTGTCATCGTCGTCCTTGTAGTCTTTGT-CATCGTCGTCCTTGTAGTCCA-3'
	Cloning	Eurofins MWG Operon	
FLAGfwd-NdeI
	5'-TTCGCCCCATATGGACTACAAGG-3'	Cloning	Eurofins MWG Operon	
FLAGrev-NdeI
	5'-TTCGCCCCATATGTTTGTCATCG-3'	Cloning	Eurofins MWG Operon	
pET24fwd-PstI
	5'-CTGCAGTAAGAAGGAGATATAC-3'	Cloning	Eurofins MWG Operon	
pET24rev-HindIII
	5'-AAGCTTTAGCAGCCGGATCTCA-3'	Cloning	Eurofins MWG Operon	
pepDstart-NheI
	5'-GCTAGCGCCAAGTTGGCCCGAGTA-3'	Cloning	Eurofins MWG Operon	
pET24fwd-PacI
	5'-TTAATTAATAAGAAGGAGATATAC-3'	Cloning	Eurofins MWG Operon	
Rv2744cFWD-EcoRI
	5'-GAATTCGCCAATCCGTTCGTTAAAGCC-3'	Cloning	Eurofins MWG Operon	
Rv2744cREV-NotI
	5'-GCGGCCGCTGACCGTAGGGGCTGCTC-3'	Cloning	Eurofins MWG Operon	
Rv2744cFWD-NheI
	5'-GCTAGCGCCAATCCGTTCGTTAAAGCC-3'	Cloning	Eurofins MWG Operon	
pET24REV-EcoRV
	5'-GATATCTAGCAGCCGGATCTCA-3'	Cloning	Eurofins MWG Operon	
Rv2744cRev4-NotI	5'-GCGGCCGCCTGACCGTAGGGCTGCTC-3'	Cloning	Eurofins MWG Operon	

Rv2744cfwd-XbaI
	
5'-TCTAGAGGCCAATCCGTTCGTTAA-3'	
Cloning	
Eurofins MWG Operon	
Rv2744crev-KpnI
	5'-GGTACCCGCTGACCGTAGGGCT-3'	Cloning	Eurofins MWG Operon	
pepDS-Afwd
	5'-GTAACGCAGGGGGCGCGCTGGTGAACATGAAC-3'	Quickchange	Eurofins MWG Operon	
pepDS-Arev2
	5'-GTTCATGTTCACCAGCGCGCCCCCTGCGTTAC-3'	Quickchange	Eurofins MWG Operon	

a. Underlined sequences in primers denote the engineered restriction sites used for cloning.
