# Supplementary material for: The HtrA-Like Serine Protease PepD Interacts with and Modulates the Mycobacterium tuberculosis 35-kDa Antigen Outer Envelope Protein
Source: PLoS One. 2011 Mar 22;6(3):e18175. doi: 10.1371/journal.pone.0018175 (PMC3062566; doi:10.1371/journal.pone.0018175)
Supplement: Table S5 — Proteins identified in both M. smegmatis cell wall and whole cell lysate preparations that co-immunoprecipitate with 3x-FLAG-PepDS317A-6xHis. (RTF) [file pone.0018175.s007.rtf]

Table S5.  Proteins identified in both M. smegmatis cell wall and whole cell lysate preparations that co-immunoprecipitate with 3xFLAG-PepDS317A-6xHisa
Accession No.	Genea	M. tuberculosis Homologueb	Found in M. tuberculosis PepD IP eluate	Gene Product	Protein Class	

MSMEG_0633
		
N/A	
No	
PAP2 superfamily protein	
Unknown function	
MSMEG_0643
		N/A	No	Extracellular solute-binding protein	Transport and binding proteins	
MSMEG_0688
		Rv0337c	No	Aspartate aminotransferase	Amino acid biosynthesis	
MSMEG_0777
		Rv0407	Yes	F420-dependent glucose-6-phosphate dehydrogenase	Energy metabolism	
MSMEG_0825
	thiD	Rv0422c	No	Phosphomethylpyrimidine kinase	Biosynthesis of cofactors	
MSMEG_1030
		N/A	No	Monooxygenase	Energy metabolism	
MSMEG_1046
		N/A	No	ABC-type molybdenum transport system, ATPase	Central intermediary metabolism	
MSMEG_1073
		Rv0547c	No	Oxidoreductase	Central intermediary metabolism	
MSMEG_1347
	rplA	Rv0641	No	50S ribosomal protein L1	Protein synthesis	
MSMEG_1353
		Rv0647c	Yes	ABC1 family protein	Unknown function	
MSMEG_1401
	tuf	Rv0685	Yes	Elongation factor Tu	Protein synthesis	
MSMEG_1424
		Rv0694	No	FMN-dependent dehyrogenase	Unknown function	
MSMEG_1435
	rpsJ	Rv0700	No	30S ribosomal protein S10	Protein synthesis	
MSMEG_1442
	rpsC	Rv0707	Yes	30S ribosomal protein S3	Protein synthesis	
MSMEG_1523
	rpsD	Rv3458c	No	30S Ribosomal protein S4	Protein synthesis	
MSMEG_1524
	rpoA	Rv3457c	Yes	DNA-directed RNA polymerase subunit alpha	Transcription	
MSMEG_1603
		Rv3410c	No	IMP dehydrogenase family protein	Unknown function	
MSMEG_1807
		Rv3285	Yes	Acetyl-/propionyl-coenzyme A carboxylase alpha chain	Fatty acid and phospholipid metabolism	
MSMEG_1937
		Rv3206c	Yes	Molybdopterin biosynthesis protein	Biosynthesis of cofactors	
MSMEG_1959
		Rv3193c	Yes	UPF0182 protein	Cell envelope	
MSMEG_2079
		N/A	No	Alcohol dehydrogenase	Energy metabolism	
MSMEG_2080
		Rv3140	Yes	Putative acyl-CoA dehydrogenase	Fatty acid and phospholipid metabolism	


MSMEG_2378	

serA
	

Rv2996c	

Yes	

D-3-phosphoglycerate dehydrogenase	

Amino acid biosynthesis	
MSMEG_2410
		Rv2969c	No	Putative serine-threonine protein kinase	Unclassified	
MSMEG_2519
	rpsB	Rv2890c	Yes	Ribosomal protein S2	Protein synthesis	
MSMEG_2613
	mqo	Rv2852c	No	Probable malate:quinone oxidoreductase	Energy metabolism	
MSMEG_2695
		Rv2744c	Yes	35 kDa protein	Protein fate	
MSMEG_3043
	pyrB	Rv1380	Yes	Aspartate carbamoyltransferase	Purines, pyrimidines, nucleosides, and nucleotides	
MSMEG_3044
		Rv1381	Yes	Dihydroorotase	Purines, pyrimidines, nucleosides, and nucleotides	
MSMEG_3084
	gap	Rv1436	Yes	Glyceraldehyde-3-phosphate dehydrogenase	Energy metabolism	
MSMEG_3124
	sufC	Rv1463	No	FeS assembly ATPase	Biosynthesis of cofactors	
MSMEG_3375
		N/A	No	Alcohol dehydrogenase	Energy metabolism	
MSMEG_3839
		Rv1629	Yes	DNA polymerase I	DNA metabolism	
MSMEG_3940
		Rv2026c	No	Universal stress protein	Cellular processes	
MSMEG_3945
		N/A	No	Universal stress protein	Cellular processes	
MSMEG_4281
		Rv2213	No	Cytosol aminopeptidase	Protein fate	
MSMEG_4283
	sucB	Rv2215	No	2-oxoglutarate dehydrogenase	Energy metabolism	
MSMEG_4327
		Rv2245	Yes	3-oxoacyl-[acyl-carrier-protein] synthase 1	Fatty acid and phospholipid metabolism	
MSMEG_4328
		Rv2246	Yes	3-oxoacyl-[acyl-carrier-protein] synthase 2	Fatty acid and phospholipid metabolism	
MSMEG_4672
	clpP	Rv2460c	No	ATP-dependent Clp protease proteolytic subunit	Protein fate	
MSMEG_4681
		N/A	No	Putative uncharacterized protein	Hypothetical proteins	
MSMEG_4722
		Rv2509	No	Short-chain dehydrogenase	Energy metabolism	
MSMEG_4916
	glgE	Rv1327c	No	Putative glucanase	Energy metabolism	
MSMEG_4936
	atpD	RV1310	No	ATP synthase subunit beta	Energy metabolism	
MSMEG_4938
	atpA	Rv1308	No	ATP synthase subunit alpha	Energy metabolism	
MSMEG_5048
		Rv1249c	No	Putative uncharacterized protein	Hypothetical proteins	


MSMEG_5225
		

Rv1109c	

No	

Putative uncharacterized protein	

Hypothetical proteins	
MSMEG_5487	mprB
	Rv0982	No	Histidine-protein kinase	Signal transduction	
MSMEG_5512
		Rv0958	No	Magnesium chelatase	Transport and binding proteins	
MSMEG_6058
		Rv3270	No	Cadmium transporting P-type ATPase	Transport and binding proteins	
MSMEG_6091
	clpC/mecB	Rv3596c	Yes	Negative regulator of competence	Cellular processes	
MSMEG_6284
		Rv3270	Yes	Cyclopropane-fatty-acyl-phospholipid synthase	Fatty acid and phospholipid metabolism	
MSMEG_6385
		Rv3791	No	Putative oxidoreductase	Energy metabolism	
MSMEG_6403
		Rv3808c	No	Bifunctional udp-galactofuranosyl transferase	Cell envelope	
MSMEG_6471
		N/A	No	Glycine/D-amino acid oxidase	Transport and binding proteins	
MSMEG_6761
		N/A	No	Glycerol-3-phosphate dehydrogenase	Energy metabolism	

a Protein information annotated based on the JCVI Comprehensive Microbial Resource website (http://cmr.jcvi.org/cgi-bin/CMR/CmrHomePage.cgi).

b No homolog in M. tuberculosis H37Rv.
