# Supplementary material for: The HtrA-Like Serine Protease PepD Interacts with and Modulates the Mycobacterium tuberculosis 35-kDa Antigen Outer Envelope Protein
Source: PLoS One. 2011 Mar 22;6(3):e18175. doi: 10.1371/journal.pone.0018175 (PMC3062566; doi:10.1371/journal.pone.0018175)
Supplement: Table S8 — Identification of putative autolytic PepD cleavage sites using LC-MS/MS. (RTF) [file pone.0018175.s010.rtf]

Table S8.  Identification of putative autolytic PepD cleavage sites using LC-MS/MS.
Scan Count	Peptide identifieda	Previously identified cleavage siteb	

149
	
A.ANMPPGSVEQVAAK	
Yes	
35
	D.PTSDIAVVR	No	
24
	P.GATVALTFQDPSGGSR	No	
24
	A.SAAPSIPAANMPPGSVEQVAAK	No	
15
	R.SKAPGATVALTFQD	No	
11
	F.TVVGADPTSDIAVVR	No	
7
	D.RPINSADALVAAVR	No	
5
	D.DRPINSADALVAAVR	No	
4
	V.PSVVMLETDLGR	No	
4
	V.VGADPTSDIAVVR	No	
3
	D.AQSGSIGLGFAIPVDQAK	No	
3
	D.SADAQSGSIGLGFAIPVDQAK	No	
2
	S.IPAANMPPGSVEQVAAK	No	
2
	S.VVMLETDLGR	No	
2
	L.GADSADAQSGSIGLGFAIPVDQAK	No	
2
	Q.PGTPGYAQGQQQTYSQQFDWR	No	
2
	V.AASAAPSIPAANMPPGSVEQVAAK	No	
2
	G.GPVAASAAPSIPAANMPPGSVEQVAAK	No	
2
	A.DALVAAVR	No	
1
	V.SGLTPISLGSSSDLR	No	
1
	V.TNDKDTLGAK	Yes	
1
	S.GGPVAASAAPSIPAANMPPGSVEQVAAK	No	
1
	V.DDRPINSADALVAAVR	No	
1
	S.ADAQSGSIGLGFAIPVDQAK	No	
1
	V.GLVQEEQPSDM*TNHPR	No	
1
	N.TVLDAIQTDAAINPGN	No	
1
	A.GPSGGPVAASAAPSIPAANMPPGSVEQVAAK	No	
1
	Q.SGSIGLGFAIPVDQAK	No	
1
	G.LGFAIPVDQAK	No	
1
	V.VMLETDLGR	No	
1
	G.PSGGPVAASAAPSIPAANMPPGSVEQVAAK	No	
1
	A.GIGGAAASLVGFNR	No	

1
	
G.LTPISLGSSSDLR	
No	
1	S.IGLGFAIPVDQAK
	No	
1	A.PFTVVGADPTSDIAVVR
	No	
1	P.AGPSGGPVAASAAPSIPAANMPPGSVEQVAAK
	No	
1	V.ALTFQDPSGGSR
	Yes	
1	L.GVQVTNDKDTLGAK
	No	
1	S.GSIGLGFAIPVDQAK
	No	
1	M.PPGSVEQVAAK
	Yes	
1	R.QPYEALGGTRPGLIPG
	No	
1	T.APFTVVGADPTSDIAVVR
	No	
1	G.SIGLGFAIPVDQAK
	No	
a Semi-tryptic peptide identification with annotated cleavage site.
b Mohamedmohaideen, et al.(2008)
